# When Doing Nothing Feels Safer: A Multilevel Framework for Therapeutic Inertia in Psychiatry

**DOI:** 10.3390/healthcare14142212

**Published:** 2026-07-21

**Authors:** Carlos De las Cuevas

**Affiliations:** 1 Instituto Universitario de Neurociencia (IUNE), University of La Laguna, 38070 San Cristóbal de La Laguna, Canary Islands, Spain; ccuevas@ull.edu.es; 2 Department of Internal Medicine, Dermatology and Psychiatry, Faculty of Medicine, University of La Laguna, 38070 San Cristóbal de La Laguna, Canary Islands, Spain

**Keywords:** therapeutic inertia, psychiatry, clinical decision-making, treatment non-modification, risk perception, shared decision-making, deprescribing, healthcare systems

## Abstract

Therapeutic inertia has been extensively examined in chronic medical conditions but remains insufficiently conceptualized and empirically studied in psychiatry. This structured narrative review examines therapeutic inertia as a multilevel and potentially bidirectional phenomenon arising when clinically relevant care is not reconsidered or modified despite unmet therapeutic goals and the availability of a reasonable and feasible alternative. A targeted PubMed search was supplemented by backward and forward citation searching, prioritizing psychiatric evidence and foundational literature on clinical decision-making under uncertainty. The review distinguishes therapeutic inertia from appropriate caution, watchful waiting, informed refusal, structural non-access, therapeutic nihilism, medical futility, therapeutic obstinacy, and evidence-based deprescribing. Potential determinants include cognitive and emotional mechanisms, diagnostic and prognostic uncertainty, adverse-effect concerns, patient preferences and previous experiences, therapeutic relationships, resource constraints, fragmented care, guideline structures, workload, and medicolegal culture. Clozapine underutilization in treatment-resistant schizophrenia represents the most compelling psychiatric example, while direct but more limited evidence is available in major depressive and bipolar disorders; applications to anxiety, obsessive-compulsive, substance-use, and non-pharmacological care remain largely hypothesis-generating. Strategies include explicit therapeutic goals, planned reassessment, measurement-based and guideline-informed care, shared decision-making, multidisciplinary review, improved access, clinical decision support, and audit and feedback. Future research should use operational definitions, experimental and observational designs, patient-centered outcomes, and real-world data to determine when treatment non-modification is inappropriate and whether corrective interventions improve care without promoting indiscriminate escalation, coercion, polypharmacy, or premature discontinuation.

## 1. Introduction

Clinical decision-making in psychiatry frequently occurs under substantial uncertainty. Although biological measures are increasingly investigated and used in selected contexts, psychiatry does not commonly employ biomarkers with sufficient individual-level accuracy to determine illness trajectory, treatment response, relapse risk, or suicidal behavior across routine practice [1]. Diagnosis and prognosis therefore continue to rely largely on phenomenological assessment, longitudinal observation, and probabilistic reasoning [2,3,4].

This uncertainty matters because both intervention and non-intervention may cause harm. Psychiatric treatments may produce adverse effects, treatment burden, withdrawal phenomena, or loss of autonomy, whereas delayed or insufficient treatment may contribute to persistent symptoms, suicidality, hospitalization, functional deterioration, chronic suffering, and premature mortality [5]. Clinicians are therefore often choosing between competing and incompletely predictable risks rather than between risk and safety.

Therapeutic inertia was originally defined in chronic medical care as failure to initiate or intensify treatment despite unmet therapeutic goals [6,7,8,9,10]. Its application to psychiatry remains comparatively underdeveloped [11], despite the relevance of diagnostic ambiguity, heterogeneous response, delayed outcomes, adverse-effect concerns, patient preferences, limited access to specialist interventions, and institutional or medicolegal pressures.

Not every unchanged treatment represents therapeutic inertia. Non-modification may reflect appropriate caution, unresolved diagnostic uncertainty, limited expected benefit, contraindication, informed refusal, patient preference, or a defined period of observation with planned reassessment. Structural barriers may also prevent implementation of an otherwise appropriate intervention. A clinically useful formulation must therefore distinguish insufficiently justified treatment persistence from appropriate non-escalation and restricted access to care.

The concept should also be bidirectional. Therapeutic inertia may involve failure to initiate, intensify, switch, or augment treatment, but also failure to simplify, taper, or discontinue an intervention that is ineffective, unnecessarily burdensome, or disproportionately harmful. This distinction is especially relevant in psychiatry, where undertreatment may coexist with polypharmacy, overtreatment, coercion, and prolonged continuation of therapies whose benefit has not been adequately reassessed.

Risk perception may contribute to treatment non-modification. Behavioral decision research suggests that harms associated with action may be perceived differently from harms associated with inaction, particularly when outcomes are emotionally salient, readily attributable, or vulnerable to retrospective scrutiny [12,13,14,15,16,17,18,19,20]. Omission bias, loss aversion, ambiguity aversion, status quo bias, and hindsight bias therefore provide plausible mechanisms, although their direct contribution to therapeutic inertia in psychiatry remains insufficiently measured.

This structured narrative review and conceptual synthesis has five objectives: to propose a precise and bidirectional definition of therapeutic inertia; to distinguish it from appropriate caution, informed refusal, structural non-access, therapeutic nihilism, medical futility, therapeutic obstinacy, and deprescribing; to examine cognitive, clinical, relational, and system-level determinants; to evaluate clinical expressions according to the strength of the available evidence; and to outline mitigation strategies and an empirical research agenda.

## 2. Methods and Scope

This article was developed as a structured narrative review and conceptual synthesis. To inform the revised manuscript, the relevant literature was reassessed and updated through a targeted search of PubMed, completed on 13 July 2026. The search combined terms related to therapeutic inertia (“therapeutic inertia”, “clinical inertia”, “treatment inertia”, “undertreatment”, and “treatment intensification”) with terms referring to psychiatry, mental disorders, and relevant clinical contexts, including schizophrenia, major depressive disorder, bipolar disorder, anxiety disorders, obsessive–compulsive disorder, substance use disorders, clozapine, electroconvulsive therapy, and psychopharmacological deprescribing. Additional searches combined psychiatric terms with “risk perception”, “omission bias”, “status quo bias”, “loss aversion”, “ambiguity aversion”, “uncertainty”, “defensive medicine”, “medicolegal”, “shared decision-making”, “patient refusal”, “medical futility”, “therapeutic nihilism”, “therapeutic obstinacy”, “resource constraints”, “measurement-based care”, and “clinical decision support”.

The reference lists of relevant reviews and key primary studies were examined, and supplementary forward citation searches were conducted to identify additional and more recent publications. Eligible sources included peer-reviewed empirical studies, systematic and narrative reviews, consensus or guideline documents, and seminal theoretical contributions relevant to the definition, determinants, clinical manifestations, consequences, or mitigation of therapeutic inertia. Priority was given to psychiatric evidence and to recent higher-level evidence when available. Literature from other medical specialties was retained when it provided foundational definitions or established models of therapeutic inertia, risk perception, or clinical decision-making under uncertainty. Sources addressing non-adherence, informed refusal, patient preference, or restricted access to care were considered when they helped distinguish therapeutic inertia from conceptually related but different causes of treatment non-modification.

Because the purpose of the review was conceptual integration rather than estimation of a pooled effect, no meta-analysis or formal risk-of-bias assessment was undertaken. The literature was synthesized thematically across clinician-, patient-, treatment-, and healthcare-system-level domains, with an explicit distinction between empirically supported observations and hypothesis-generating propositions. The reporting of the review was informed by the quality domains of the Scale for the Assessment of Narrative Review Articles (SANRA) [21]. This approach does not constitute a systematic review and does not claim exhaustive retrieval of all potentially relevant publications.

## 3. Defining Therapeutic Inertia and Its Conceptual Boundaries

The concept of therapeutic inertia originated in chronic medical care and was initially defined as failure to initiate or intensify treatment despite unmet therapeutic goals [6]. Later models recognized that treatment non-modification may arise from interacting clinician-, patient-, treatment-, and healthcare-system-related factors rather than from inadequate knowledge or professional neglect alone [7,8,9,10]. In psychiatry, the concept requires particular precision because maintaining treatment may reflect appropriate caution, diagnostic uncertainty, limited expected benefit, patient preference, treatment refusal, contraindication, or restricted access to care.

For the purposes of this review, therapeutic inertia in psychiatry is defined as a clinically consequential and insufficiently justified delay or failure to reconsider or modify treatment when a relevant therapeutic goal remains unmet and a reasonable and feasible alternative is available. Modification may involve initiation, intensification, switching, augmentation, simplification, tapering, discontinuation, or introduction of a non-pharmacological intervention. The definition is therefore bidirectional and should not be equated with failure to prescribe more treatment.

The designation becomes more defensible when treatment resistance is established, clinically significant symptoms, disability, or suicide risk persist, no overriding contraindication exists, and the decision is not primarily explained by informed patient preference, refusal, contraindication, unresolved diagnostic uncertainty, limited expected benefit, or practical unavailability. These conditions constitute a conceptual proposal rather than a validated instrument.

Appropriate clinical caution differs from therapeutic inertia because it is reasoned, proportionate, time-limited, and accompanied by explicit criteria for review. Watchful waiting may be justified when symptoms are mild or fluctuating, spontaneous improvement is plausible, the diagnosis remains uncertain, or treatment risks outweigh likely short-term benefits. The distinction depends less on whether treatment changes than on whether the decision is transparent, evidence-informed, concordant with the patient’s goals, and subject to planned reassessment.

Patient preference, shared decision-making, and informed refusal must likewise be distinguished from clinician inaction. A competent patient may reasonably decline, postpone, reduce, or discontinue treatment after receiving balanced information about the likely consequences of both intervention and non-intervention [22,23,24,25,26,27]. Apparent reluctance, however, should not be assumed to represent autonomous preference when alternatives have been inadequately explained or practical support has not been offered.

Structural barriers require separate attribution. Lack of access to clozapine monitoring, electroconvulsive therapy, specialist consultation, psychotherapy, rehabilitation, or substance-use treatment should not automatically be described as individual clinician inertia. When organizations repeatedly fail to address known and remediable barriers despite clinically consequential delay, a system-level form of therapeutic inertia may be a more appropriate formulation [25,26,27,28].

Therapeutic inertia also differs from therapeutic nihilism, medical futility, and therapeutic obstinacy. Therapeutic nihilism refers to a prematurely pessimistic belief that meaningful improvement is unlikely and may act as an antecedent of inertia [29]. Medical futility concerns interventions judged unlikely to achieve a meaningful therapeutic objective; proportionate non-escalation in such circumstances should not be labelled inertia, although prognostic uncertainty makes claims of futility especially difficult in psychiatry [30]. Therapeutic obstinacy describes persistence or escalation despite very low expected benefit or disproportionate burden [31]. Both inertia and obstinacy may therefore involve failure to revise an established treatment strategy when the balance of benefits, harms, and patient priorities has changed.

Deprescribing should not be framed as intrinsically better or worse than intensification. Appropriate deprescribing is an active, planned, and supervised process intended to reduce unnecessary treatment burden or disproportionate harm [32]. Failure to discontinue an ineffective or harmful intervention may itself represent therapeutic inertia. Conversely, poorly planned withdrawal may increase the risk of recurrence, withdrawal phenomena, or relapse. In stable schizophrenia-spectrum disorders, discontinuation or substantial dose reduction is associated on average with greater relapse risk than continuation at standard doses, although individualized reduction may be appropriate [33]. Antidepressant deprescribing similarly requires consideration of previous episodes, residual symptoms, adverse effects, patient preference, withdrawal risk, and relapse monitoring [34].

Therapeutic inertia should therefore be understood neither as a synonym for caution nor as an argument for greater treatment intensity. It describes failure to reconsider or modify care when modification has become clinically warranted and maintaining the status quo is no longer adequately justified. Table 1 summarizes the principal distinctions between therapeutic inertia and related forms of appropriate non-modification, structural non-access, pessimistic therapeutic attitudes, excessive treatment persistence, and deprescribing.

## 4. Multilevel Determinants of Therapeutic Inertia in Psychiatry

Therapeutic inertia should not be attributed solely to deficient knowledge, inadequate motivation, or individual clinician failure. Models developed in other chronic conditions increasingly describe treatment non-modification as the result of interacting clinician-, patient-, treatment-, and healthcare-system-level determinants [7,8,9,10]. This perspective is particularly relevant to psychiatry, where decisions frequently involve diagnostic uncertainty, heterogeneous treatment response, preference-sensitive outcomes, substantial adverse-effect burdens, and organizational constraints. Direct psychiatric evidence remains limited, however, and several mechanisms discussed below should be regarded as plausible and hypothesis-generating rather than established causes [11].

### 4.1. Cognitive and Emotional Mechanisms

Clinical judgments are influenced not only by estimated probabilities of benefit and harm, but also by framing, emotional salience, anticipated regret, perceived responsibility, previous experience, and the availability of memorable adverse outcomes [12,13,14,15,16,17,18,19]. These influences are ordinary features of decision-making and may become more consequential when evidence is incomplete and outcomes are emotionally charged.

Omission bias may favor harms arising from inaction over comparable harms attributable to intervention, while status quo bias may support continuation of an established plan because changing course requires additional psychological and operational justification [14,35]. Loss aversion and ambiguity aversion may further discourage intervention when potential harms are concrete and benefits remain uncertain [15,16,17]. Although these biases are well described in decision-making research, their direct contribution to therapeutic inertia in psychiatry has not been adequately quantified.

Psychiatric adverse outcomes may differ in visibility and attribution. A severe medication reaction, compulsory intervention, or suicide following treatment modification may be experienced as closely linked to an active decision, whereas persistent symptoms or functional decline during unchanged treatment may be less clearly attributable to a single choice. Hindsight bias may intensify this asymmetry by making adverse outcomes appear more predictable retrospectively than they were prospectively [20].

Cognitive bias does not invariably favor inaction. Commission bias, therapeutic enthusiasm, and discomfort with persistent symptoms may encourage unnecessary escalation, coercion, or polypharmacy. The relevant issue is therefore not a general tendency toward passivity, but distortion in the proportional comparison of competing risks.

### 4.2. Clinical and Treatment-Related Factors

Psychiatric treatment decisions often rely on probabilistic clinical formulations. Although biological measures are increasingly investigated and used in selected contexts, biomarkers with sufficient individual-level accuracy are not commonly available to determine diagnosis, prognosis, relapse, treatment response, or suicide risk across routine practice [1,2,3,4]. Diagnostic boundaries may remain uncertain, comorbidity is frequent, and symptom trajectories may fluctuate independently of treatment.

Response is also often delayed, partial, or multidimensional. Symptom improvement may coexist with disability, poor quality of life, adverse effects, or failure to achieve outcomes valued by the patient. Without explicit goals and scheduled reassessment, partial response may gradually become accepted without a deliberate judgment that it is sufficient.

Concern about adverse effects may appropriately delay treatment modification. Clozapine, lithium, electroconvulsive therapy, augmentation strategies, deprescribing, and long-term pharmacotherapy may require careful selection, communication, and monitoring [36,37,38,39]. Hesitation becomes potentially inertial when it remains open-ended, no alternative plan is developed, and no criteria are defined for reconsideration.

Suicide risk illustrates the difficulty of balancing competing harms. Clinicians must respond to potentially lethal risk despite limited individual-level predictive accuracy [40]. Both insufficient intervention and unnecessarily restrictive or coercive treatment may cause harm. The relevant safeguard is proportionate reasoning, collaborative planning, and longitudinal reassessment rather than automatic escalation or avoidance.

Clinical familiarity may also reduce perceived urgency. Persistent psychosis, residual depression, chronic anxiety, negative symptoms, or functional impairment may become incorporated into expectations about the patient’s usual condition. This normalization is a plausible mechanism, particularly after repeated treatment failures, but should not be assumed to characterize psychiatric practice generally.

### 4.3. Patient and Relational Factors

Patient-related determinants should not be framed as isolated deficits. Treatment decisions are shaped by preferences, previous experiences, beliefs about illness and treatment, trust, social circumstances, available support, and practical capacity to undertake care. Psychiatric symptoms may affect motivation, concentration, hope, insight, or decisional capacity, but such effects vary and should not be inferred solely from diagnosis.

Patients and clinicians may also prioritize different outcomes. Clinicians may emphasize symptoms, relapse, or hospitalization, whereas patients may assign greater importance to cognitive clarity, emotional experience, autonomy, sexual functioning, weight, employment, relationships, or freedom from monitoring. Apparent non-escalation may therefore be reasonable when these priorities are considered.

Treatment refusal should not be labelled therapeutic inertia. Clinicians must nevertheless ensure that alternatives and the consequences of both intervention and non-intervention are communicated comprehensibly, that modifiable barriers are addressed, and that decisions remain open to reconsideration. Shared and supported decision-making may facilitate this process, although implementation is limited by relational, organizational, and risk-management barriers [22,23,24,25].

Relational dynamics may contribute in both directions. Difficult decisions may be repeatedly deferred to preserve a fragile alliance or avoid conflict, while intervention may sometimes proceed too rapidly without adequate exploration of the patient’s priorities. A patient-centered model must therefore protect against both therapeutic abandonment and paternalistic over-intervention.

### 4.4. Organizational, Structural, and Institutional Factors

Treatment decisions depend on what healthcare systems can practically provide. Restricted access to clozapine monitoring, electroconvulsive therapy, specialist consultation, psychotherapy, rehabilitation, supported housing, or substance-use services may delay appropriate care independently of an individual clinician’s intentions [28,36,37]. Waiting lists, geographical inequalities, fragmented services, insufficient staffing, and poor continuity may further impede reassessment and implementation.

Some interventions require substantial infrastructure and coordination. When services cannot provide this support, apparent clinical non-intervention may reflect structural non-access rather than clinician inertia. Repeated organizational failure to address known and remediable barriers may nevertheless constitute a system-level form of therapeutic inertia [41].

Time and economic pressures may also favor continuation of the status quo. Short consultations, high caseloads, productivity targets, administrative burden, and fragmented responsibility can make reassessment, shared decision-making, monitoring, and multidisciplinary planning difficult [9,28,36,42]. Burnout may further reduce clinicians’ capacity to engage with complex and uncertain decisions, although workload and burnout are themselves organizational phenomena rather than individual failings [42].

Clinical guidelines may reduce delay by defining treatment targets, adequate trials, monitoring, and reassessment intervals. Conversely, complex eligibility requirements, monitoring burdens, or disproportionate emphasis on intervention-related risks could reinforce conservative practice [7,9,11]. Whether this occurs in particular psychiatric guidelines requires empirical study.

Institutional and medicolegal cultures may also influence clinical thresholds. Defensive practice is shaped by liability concerns, professional norms, documentation requirements, organizational accountability, and anticipated scrutiny [43,44]. In psychiatry, adverse outcomes involving suicide, violence, coercion, or severe treatment-related harm may attract particularly close review [45]. These pressures may improve accountability but could also favor decisions perceived as easier to defend retrospectively.

Institutional incentives do not always promote inaction. Protocols, performance targets, and risk-management practices may also encourage unnecessary hospitalization, coercion, medication, or polypharmacy. Systems can therefore contribute to both undertreatment and overtreatment. The central question is whether they support individualized, proportionate, and revisable decisions rather than whichever option appears least institutionally vulnerable.

**Figure 1:**
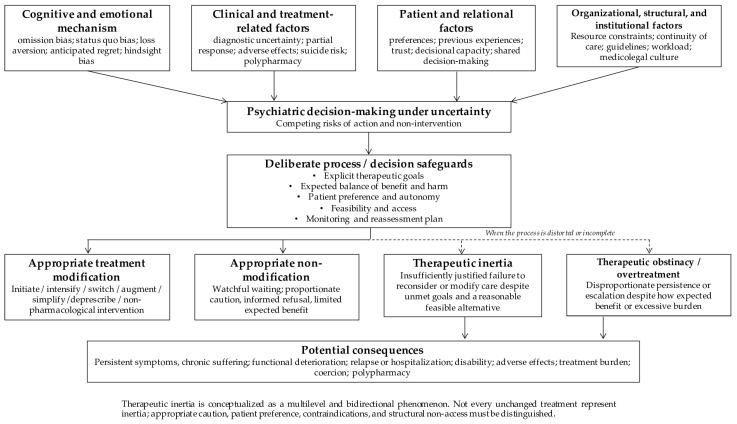
Multilevel framework for therapeutic decision-making and therapeutic inertia in psychiatry. Cognitive, clinical, patient-related, relational, organizational, and institutional determinants converge in psychiatric decision-making under uncertainty. A deliberative process incorporating explicit therapeutic goals, proportional assessment of benefits and harms, patient preferences, feasibility, and planned reassessment may support either appropriate treatment modification or justified non-modification. When this process is incomplete or distorted, treatment may remain insufficiently reconsidered despite unmet goals—therapeutic inertia—or may persist or escalate despite limited expected benefit or disproportionate burden—therapeutic obstinacy. The model is conceptual and hypothesis-generating.

Therapeutic inertia is thus best understood as a multilevel process in which cognitive mechanisms, clinical uncertainty, patient priorities, therapeutic relationships, available resources, and institutional incentives interact. Their relative influence is likely to vary across disorders, treatments, settings, and healthcare systems. Figure 1 summarizes this conceptual framework.

## 5. Clinical Expressions and Strength of Evidence Across Psychiatric Disorders

The evidence for therapeutic inertia is uneven across psychiatric disorders. Three levels should be distinguished: direct studies that operationalize treatment non-modification despite predefined indications for review; documented delays or underuse despite persistent nonresponse and an available evidence-based alternative; and broader treatment gaps that may reflect inertia but also non-recognition, patient preference, contraindication, stigma, or structural non-access. The examples below are therefore presented according to their relative evidential strength rather than as equivalent manifestations of a single phenomenon.

### 5.1. Schizophrenia-Spectrum Disorders and Clozapine

Clozapine underutilization in treatment-resistant schizophrenia remains the most compelling psychiatric example of possible therapeutic inertia. Its efficacy in appropriately defined treatment resistance is well established, yet delayed initiation and underprescription have been documented across healthcare systems [36,37,46,47,48,49]. Treatment-trajectory studies show that some patients receive several ineffective antipsychotic trials before clozapine is considered [46,47]. Clozapine also has specific evidence for reducing suicidal behavior in schizophrenia and schizoaffective disorder [50].

These delays have heterogeneous causes. Concerns about neutropenia, myocarditis, pneumonia, seizures, metabolic burden, monitoring, adherence, and discontinuation are legitimate [36,37,49,51]. Patient refusal, contraindications, previous adverse experiences, limited clinician familiarity, and unavailable monitoring may also contribute. Underuse at the population level therefore does not establish inertia in every case.

The designation becomes more defensible when treatment resistance is established, clinically significant symptoms, disability, or suicide risk persist, no overriding contraindication exists, and clozapine is neither discussed nor replaced by a documented alternative plan. By contrast, informed refusal or inability to provide safe monitoring should be classified as preference-sensitive non-initiation or structural non-access.

The bidirectional framework is also relevant. Failure to consider clozapine, psychosocial interventions, or rehabilitation may represent undertreatment, whereas repeated augmentation, high-dose treatment, or persistent polypharmacy without meaningful benefit may represent therapeutic obstinacy or inertia in the direction of treatment persistence.

### 5.2. Depressive and Bipolar Disorders

Major depressive disorder provides emerging direct evidence. Real-world studies have identified patients whose treatment remained unchanged despite persistent symptoms and unmet treatment goals [38]. Structured algorithms and measurement-based care may help make nonresponse visible and link it to explicit decisions about optimization, switching, augmentation, psychotherapy, or referral [39,52].

Inertia in depression may involve prolonged continuation of a partially effective treatment without explicit reassessment of residual symptoms, functioning, adverse effects, or suicide risk. Repeated postponement of electroconvulsive therapy, lithium augmentation, or specialist review could also represent inertia when the indication is clear and delay is not explained by preference, contraindication, or feasibility. These examples are less straightforward than clozapine because later-line sequencing in depression is more heterogeneous.

The concept is also bidirectional. Failure to discontinue an ineffective antidepressant, accumulation of poorly justified combinations, or neglect of burdensome adverse effects may constitute treatment persistence rather than appropriate continuity. Antidepressant deprescribing should therefore be individualized according to previous episodes, residual symptoms, duration of remission, withdrawal risk, adverse effects, patient preference, and relapse monitoring [34].

Bipolar disorder is one of the few areas in which clinical inertia has been explicitly operationalized. In the STEP-BD study, at least one indication for medication adjustment was identified in 36% of visits, and no adjustment occurred in 19% of those visits, most commonly despite nonresponse, adverse effects, or a new mood episode [53]. These findings support clinically relevant non-modification, although they do not establish that every unchanged regimen was inappropriate because the reasons for individual decisions were incompletely captured.

### 5.3. Anxiety Disorders and Obsessive–Compulsive Disorder

Direct evidence in anxiety disorders is limited. World Mental Health Survey data show substantial treatment and adequacy gaps, but such findings cannot distinguish clinician inertia from non-recognition, limited perceived need, patient-level barriers, or restricted access [54].

Possible inertia after diagnosis may include prolonged ineffective treatment without response monitoring, repeated symptomatic prescribing without access to disorder-specific psychotherapy, or failure to reconsider burdensome long-term treatment. These remain hypothesis-generating applications rather than established prevalence estimates.

Obsessive–compulsive disorder provides clearer evidence of clinically important delay, although not necessarily clinician-level inertia. Untreated illness may persist for years, and longer delays are associated with greater disability and poorer outcomes [55]. Concealment, stigma, misdiagnosis, limited recognition, patient reluctance, and poor access to specialized psychotherapy all contribute. The concept of inertia is most applicable after diagnosis, when clinically significant symptoms persist and evidence-based treatment or specialist referral is repeatedly deferred without proportionate justification or a reassessment plan.

### 5.4. Substance-Use Disorders

Substance-use disorders illustrate the distinction between therapeutic inertia and broader implementation failure. Effective pharmacological and psychosocial treatments remain underused. In a Swedish national cohort of adults with alcohol use disorder, approximately 23% received an approved pharmacotherapy during the following year, with substantial sociodemographic and clinical inequalities [56].

Low treatment uptake alone does not establish inertia. Access, service fragmentation, stigma, clinician training, comorbidity, patient goals, contraindications, and preference for psychosocial or harm-reduction approaches may all influence treatment. Possible inertia may occur when repeated relapse, medical harm, or expressed willingness to change does not prompt discussion of pharmacotherapy, psychosocial treatment, harm reduction, or referral. Conversely, rigid continuation of an unwanted or ineffective intervention may represent overtreatment.

### 5.5. Non-Pharmacological and Recovery-Oriented Care

Therapeutic inertia should not be restricted to medication. Delays in psychotherapy, electroconvulsive therapy, occupational rehabilitation, supported employment, family intervention, cognitive remediation, housing support, and integrated substance-use care may contribute to persistent disability. Direct evidence identifying these delays specifically as therapeutic inertia remains limited, and structural non-access may often be the more appropriate explanation.

Across disorders, the central question is not whether treatment was intensified, but whether persistent symptoms, disability, risk, adverse effects, or treatment burden prompted an explicit, proportionate, and patient-centered reconsideration of available options. Table 2 summarizes the evidential status and main interpretive limitations of these clinical domains.

## 6. Strategies to Reduce Therapeutic Inertia

No single intervention is likely to reduce therapeutic inertia because treatment non-modification arises from interacting clinical, relational, and organizational factors. Moreover, corrective strategies should not simply increase treatment intensity. Their purpose should be to ensure that persistent symptoms, disability, risk, adverse effects, and treatment burden prompt explicit reconsideration of continuation, intensification, switching, simplification, deprescribing, or non-pharmacological care.

### 6.1. Explicit Goals and Planned Reassessment

Therapeutic goals should be defined prospectively and extend beyond symptom reduction to functioning, quality of life, adverse effects, treatment burden, relapse prevention, and outcomes prioritized by the patient. Expected time course, monitoring, and criteria for reconsideration should also be specified.

Scheduled review points may prevent a reasonable period of observation from becoming indefinite treatment persistence. At each review, clinicians should distinguish adequate response, acceptable partial response, nonresponse, intolerance, and unresolved uncertainty. An unchanged regimen should therefore remain an explicit and documented decision rather than the default consequence of continuity.

### 6.2. Measurement-Based and Guideline-Informed Care

Measurement-based care may make persistent symptoms, functional impairment, adverse effects, or deterioration more visible and reduce reliance on retrospective global impressions. In depressive disorders, randomized and meta-analytic evidence suggests possible benefits for symptom and remission outcomes and for timely treatment adjustment, although findings remain heterogeneous and cannot be generalized to all disorders or settings [52,57].

Measurement should support rather than replace clinical interpretation. Numerical nonresponse does not automatically require pharmacological escalation, and standardized measures should be considered alongside functioning, adverse effects, preferences, and context.

Guidelines may reduce delay by defining adequate trials, indications, monitoring, and reassessment intervals. Conversely, complex eligibility criteria, monitoring burdens, or disproportionate emphasis on intervention-related harms could reinforce conservative practice. Guideline-concordant care should therefore remain individualized and attentive to both undertreatment and overtreatment.

### 6.3. Shared Decision-Making and Multidisciplinary Review

Shared decision-making can help distinguish inertia from informed and preference-concordant non-modification. Discussion should include not only the risks of intervention, but also the foreseeable consequences of persistent illness, treatment delay, or continuation of a burdensome and insufficiently effective therapy [22,23,24,25,26,27].

Complex or treatment-resistant cases may benefit from structured multidisciplinary review involving psychiatry, psychology, nursing, pharmacy, primary care, social work, occupational therapy, or specialist services. Such review should lead to a documented decision, clear responsibility, and a defined follow-up plan rather than additional administrative delay.

Patient education and decision aids may improve risk communication when they present benefits, burdens, uncertainties, and the option of continued treatment in a balanced manner. Their role is to support deliberation, not to steer patients toward a predetermined choice.

### 6.4. Access and Organizational Responsibility

Individual reflection cannot compensate for unavailable treatment. Healthcare systems should ensure practical access to clozapine monitoring, electroconvulsive therapy, evidence-based psychotherapy, rehabilitation, supported employment, and integrated substance-use care. Relevant indicators include waiting time, time from eligibility to treatment offer, number of unsuccessful treatments before specialist intervention, and inequalities in access.

Fragmented care may allow reassessment to be repeatedly deferred because responsibility is unclear. Referral should not be considered completion of the therapeutic process when the recommended intervention remains inaccessible or unimplemented.

Organizations should also examine whether consultation length, workload, productivity targets, administrative burden, or monitoring requirements make continuation of ineffective treatment easier than therapeutic change. Potential responses include protected time for complex review, specialist pathways, pharmacist-supported monitoring, rapid consultation, and clearer escalation procedures.

### 6.5. Decision Support, Audit, and Professional Education

Clinical decision-support systems may identify persistent nonresponse, overdue monitoring, prolonged ineffective treatment, or eligibility for specialist intervention. Current evidence in mental healthcare remains limited, and implementation may be affected by workflow disruption, alert fatigue, poor interoperability, and limited professional trust [58]. Decision support should therefore prompt review rather than determine treatment automatically.

Audit and feedback may evaluate time to treatment modification, delay in clozapine initiation, persistence of ineffective polypharmacy, access to psychological care, or documentation of shared decision-making. Such indicators must be interpreted carefully because rewarding treatment change without assessing appropriateness could encourage indiscriminate escalation or premature discontinuation.

Professional education should address uncertainty, cognitive bias, risk communication, deprescribing, and treatment-resistant illness. Education alone will not correct structural barriers, but it may help clinicians recognize when legitimate caution has become indefinite avoidance or when treatment persistence has become disproportionate.

Institutional review should also be symmetrical and non-punitive. Adverse-outcome analyses should examine both intervention-related harm and the possible contribution of delayed, inaccessible, or insufficient treatment, with the aim of improving future decisions rather than assigning retrospective blame.

## 7. Empirical Research Agenda and Future Directions

Therapeutic inertia in psychiatry requires empirical study before its prevalence, determinants, and consequences can be estimated confidently. Research should use operational definitions that distinguish inappropriate non-modification from informed preference, contraindication, diagnostic uncertainty, justified observation, limited expected benefit, and structural non-access. Its bidirectional nature should also be recognized by measuring failure to simplify or discontinue ineffective or harmful treatment alongside failure to initiate or intensify appropriate care.

### 7.1. Testable Hypotheses and Study Designs

Experimental vignette studies could test whether clinicians respond differently when clinically comparable harms are framed as consequences of action or inaction. Outcomes might include treatment choice, perceived risk, confidence, anticipated regret, and attribution of responsibility. Discrete-choice experiments could quantify the relative influence of expected efficacy, adverse effects, monitoring burden, patient preference, medicolegal concern, and institutional support.

Prospective cohorts should examine whether predefined nonresponse, functional impairment, adverse effects, or suicide risk lead to reassessment and treatment modification within clinically relevant periods. Such studies must record the reasons for maintaining treatment, patient preferences, contraindications, availability of alternatives, and plans for follow-up; without this information, unchanged care cannot reliably be classified as inertia.

Electronic health records, registries, and prescribing databases could be used to examine treatment trajectories, including time to clozapine initiation, number of ineffective treatment trials, persistence of polypharmacy, access to specialist care, deprescribing, hospitalization, relapse, adverse effects, and functional outcomes. Multilevel analyses may help distinguish patient-, clinician-, service-, and healthcare-system-level variation.

Qualitative and mixed-methods studies involving patients, clinicians, families, pharmacists, and service managers are also needed. They may identify relational, ethical, emotional, and organizational mechanisms not captured by routine data, including fear of damaging the therapeutic alliance, previous traumatic treatment experiences, professional responsibility, institutional blame, and competing definitions of meaningful improvement.

Pragmatic and cluster-randomized trials could evaluate measurement-based care, multidisciplinary review, decision aids, electronic prompts, specialist pathways, or audit and feedback. Success should not be defined merely by producing more treatment changes. Outcomes should include appropriateness of modification, symptoms, functioning, quality of life, adverse effects, treatment burden, patient involvement, relapse, and unintended escalation or discontinuation.

Table 3 summarizes a possible empirical framework.

### 7.2. Artificial Intelligence, Precision Psychiatry, and Real-World Evidence

Artificial intelligence and predictive analytics may eventually help identify persistent nonresponse, unusual treatment trajectories, and cases warranting clinical review [59]. Precision-psychiatry models could integrate clinical history, symptoms, comorbidity, previous treatment response, adverse effects, patient-reported outcomes, and biological or digital measures.

These applications remain investigational. Performance within a development dataset does not ensure validity across populations or healthcare systems, and current machine-learning studies of treatment selection illustrate both promise and limited clinical applicability [60]. Models may also reproduce disparities, prescribing habits, and structural deficiencies present in their training data.

Real-world evidence may complement predictive approaches by identifying delays, treatment sequences, and unwarranted variation across services [38,47]. However, observational findings are vulnerable to confounding by indication, illness severity, adherence, access, and patient preference. Future studies should combine robust causal methods with clinically detailed information explaining why treatment was or was not modified.

AI, predictive analytics, and real-world data should therefore support rather than replace clinical judgment and shared decision-making. Their value lies in making patterns and uncertainties more visible, not in automating value-sensitive decisions. A system that detects nonresponse but ignores patient priorities, feasibility, or the harms of overtreatment could reproduce rather than reduce therapeutic inertia.

## 8. Conclusions

Therapeutic inertia in psychiatry should not be understood simply as failure to prescribe more treatment. It is better conceptualized as a clinically consequential and insufficiently justified failure to reconsider or modify care when therapeutic goals remain unmet and a reasonable and feasible alternative is available. Modification may involve initiation or intensification, but also switching, simplification, deprescribing, or introduction of a non-pharmacological intervention.

Not every unchanged treatment represents inertia. Appropriate caution, watchful waiting, diagnostic uncertainty, limited expected benefit, contraindication, informed refusal, patient preference, and structural non-access may all justify non-modification. The distinction depends on whether the decision is proportionate, transparent, patient-centered, and accompanied by explicit monitoring and reassessment.

The evidence remains uneven. Clozapine underutilization in treatment-resistant schizophrenia provides the most compelling psychiatric example, while direct but more limited evidence exists in depressive and bipolar disorders. In other clinical domains, treatment gaps and delays should not be attributed to therapeutic inertia without distinguishing clinician hesitation from non-recognition, patient preference, contraindication, stigma, and restricted access.

Therapeutic inertia is likely to be a multilevel and bidirectional phenomenon. Cognitive and emotional mechanisms may interact with clinical uncertainty, patient priorities, therapeutic relationships, organizational capacity, economic pressures, guidelines, and medicolegal culture. These mechanisms remain insufficiently measured in psychiatric settings and require empirical testing.

The objective is not to make psychiatry less cautious, but to make caution proportionate, explicit, patient-centered, and revisable—and equally attentive to the harms that may follow from action and from inaction.

## Figures and Tables

**Table 1:** Distinguishing therapeutic inertia from related clinical and ethical concepts.

Concept	Defining Feature	Status of Treatment Non-Modification	Role of Patient Preference and Reassessment	Illustrative Psychiatric Example
Therapeutic inertia	Clinically consequential and insufficiently justified failure to reconsider or modify treatment when relevant goals remain unmet and a reasonable alternative is available	Potentially inappropriate	Patient preferences, feasibility, contraindications, and diagnostic uncertainty must be considered; absence of change lacks an adequate rationale or reassessment plan	Repeated postponement of clozapine consideration despite established treatment resistance and ongoing clinically significant symptoms, without contraindication, informed refusal, or a documented reassessment plan
Appropriate clinical caution or watchful waiting	Deliberate, proportionate, and time-limited non-modification under uncertainty	Potentially appropriate	Goals, monitoring, duration, and criteria for reconsideration are explicit and discussed with the patient	Deferring medication change while clarifying whether emerging affective symptoms reflect bipolar disorder, substance use, or a transient reaction
Shared decision-making or informed refusal	Treatment is not initiated or is discontinued following a genuine deliberative process and an informed patient preference	Ethically and clinically appropriate when decisional requirements are met	Patient autonomy is central; information includes the foreseeable consequences of both intervention and non-intervention; the decision remains reviewable	A patient declines clozapine after balanced discussion of expected benefits, adverse effects, monitoring requirements, and alternatives
Structural non-access	A clinically relevant intervention cannot be implemented because resources, specialist services, monitoring, or organizational pathways are unavailable	Not primarily attributable to individual clinician inertia	Patient and clinician may agree on the intervention, but the system cannot provide it	ECT is indicated and accepted but unavailable because of prolonged waiting lists or lack of specialist facilities
Therapeutic nihilism	Premature or generalized belief that meaningful improvement is unlikely or impossible	May contribute to inappropriate non-modification	Patient goals and recovery potential may be underestimated; reassessment is often absent or pessimistically framed	Chronic negative symptoms or disability are accepted as immutable without reconsidering psychosocial rehabilitation or specialist treatment
Medical futility	An intervention is considered very unlikely to achieve a meaningful therapeutic objective or its burden is disproportionate to the expected benefit	Non-escalation may be appropriate	Judgments are partly value-dependent and require transparent discussion of goals, uncertainty, and alternatives	Deciding not to pursue another burdensome intervention when previous adequate trials have failed and expected benefit is extremely limited
Therapeutic obstinacy	Treatment is continued or escalated despite very low expected benefit or disproportionate burden	Potentially inappropriate persistence or escalation	Patient priorities and harms are insufficiently integrated; treatment is continued without meaningful reassessment	Continued high-dose antipsychotic polypharmacy despite lack of benefit and substantial metabolic or cognitive adverse effects
Evidence-based deprescribing	Planned and supervised dose reduction or discontinuation when treatment is no longer beneficial, is excessively burdensome, or no longer accords with the patient’s goals	Appropriate therapeutic modification when individualized and monitored	Patient preference, relapse risk, withdrawal phenomena, prior course, and follow-up planning are central	Gradual antidepressant tapering after sustained remission following individualized assessment and agreement
Inappropriate or unsupported deprescribing	Reduction or discontinuation without adequate indication, shared planning, tapering, or relapse monitoring	Potentially harmful intervention	Patient may not be adequately informed or supported; reassessment and contingency planning are insufficient	Rapid antipsychotic withdrawal in chronic psychosis without individualized relapse-risk assessment or follow-up

Note: These categories may overlap in clinical practice. Their distinction depends on the quality of the decision-making process, the proportionality of the expected benefits and harms, the availability of alternatives, patient participation, and the presence of an explicit monitoring and reassessment plan.

**Table 2:** Illustrative clinical domains and relative evidential status of therapeutic inertia in psychiatry.

Clinical Domain	Potential Expression	Relative Evidential Status	Principal Interpretive Limitation
Treatment-resistant schizophrenia	Delayed or absent consideration of clozapine after persistent nonresponse	Relatively strong evidence of underuse and delayed treatment pathways	Patient refusal, contraindications, monitoring requirements, and structural access must be excluded
Major depressive disorder	Failure to modify treatment despite persistent symptoms or unmet goals	Direct but still limited real-world evidence; supportive algorithm-based literature	Treatment targets and sequencing are heterogeneous; partial response may sometimes justify continuation
Bipolar disorder	No medication adjustment despite nonresponse, adverse effects, or a new mood episode	Direct observational operationalization of clinical inertia	Reasons for individual non-modification and patient–clinician deliberation were incompletely captured
Anxiety disorders	Continued ineffective care or failure to access disorder-specific treatment	Predominantly indirect evidence from treatment and adequacy gaps	Population treatment gaps cannot distinguish clinician inertia from recognition, preference, or access barriers
Obsessive–compulsive disorder	Delayed evidence-based treatment or specialist referral after recognition	Evidence of prolonged untreated illness and adverse consequences of delay	Much of the delay precedes diagnosis or reflects concealment and limited specialist access
Substance-use disorders	Failure to offer pharmacotherapy, psychosocial care, harm reduction, or referral despite ongoing harm	Evidence of marked treatment underuse and unequal provision	Treatment uptake is strongly affected by patient goals, stigma, service organization, and availability
Psychosocial and rehabilitative care	Delayed psychotherapy, rehabilitation, supported employment, or social intervention	Primarily conceptual and hypothesis-generating	Structural non-access may be more important than individual clinician inertia

Note: The categories represent a qualitative appraisal of the type and directness of the available literature and should not be interpreted as a formal evidence-grading exercise.

**Table 3:** Proposed empirical approaches to therapeutic inertia in psychiatry.

Research Question	Suggested Design	Key Exposure or Intervention	Potential Outcomes
Are intervention-related harms weighted more heavily than equivalent harms of non-intervention?	Randomized clinical-vignette experiment	Action-versus-inaction risk framing	Treatment choice, perceived risk, responsibility, confidence, anticipated regret
Which treatment attributes most strongly influence non-modification?	Discrete-choice experiment	Efficacy, adverse effects, monitoring, patient preference, legal risk, resource availability	Relative attribute weights and heterogeneity across stakeholders
How frequently does treatment remain unchanged after predefined nonresponse or intolerance?	Prospective cohort study	Persistent symptoms, impairment, risk, or adverse effects	Time to reassessment, modification, documented rationale, clinical outcome
How do clinicians and healthcare systems contribute independently to delay?	Multilevel retrospective cohort or registry study	Clinician, service, and system characteristics	Time to clozapine, ECT, specialist referral, deprescribing, or treatment modification
Why is an apparently indicated treatment change deferred?	Qualitative interviews or mixed-methods study	Experiences of patients, clinicians, families, and managers	Relational, cognitive, ethical, and structural explanations
Can structured care reduce inappropriate treatment persistence?	Pragmatic or cluster-randomized trial	Measurement-based care, decision support, multidisciplinary review, audit and feedback	Appropriate treatment modification, symptoms, functioning, adverse effects, patient involvement
Can predictive models identify likely inertia without promoting overtreatment?	Prospective external validation and impact study	AI or clinical prediction model	Calibration, discrimination, fairness, clinical usefulness, treatment burden, unintended consequences

Note: Classification of treatment non-modification as therapeutic inertia requires information about clinical indication, patient preference, feasibility, contraindications, and the rationale and plan for reassessment.

## Data Availability

No new data were created or analyzed in this study. Data sharing is not applicable to this article.
